# Mortality is increased in patients with rheumatoid arthritis or diabetes compared to the general population – the Nord-Trøndelag Health Study

**DOI:** 10.1038/s41598-020-60621-2

**Published:** 2020-02-27

**Authors:** Ingrid Sæther Houge, Mari Hoff, Ranjeny Thomas, Vibeke Videm

**Affiliations:** 1 Department of Clinical and Molecular Medicine, NTNU – Norwegian University of Science and Technology, Trondheim, Norway; 2 Department of Public Health and Nursing, NTNU – Norwegian University of Science and Technology, Trondheim, Norway; 3 Department of Rheumatology, St. Olavs University Hospital, Trondheim, Norway; 4 Diamantina Institute, University of Queensland, Brisbane, Australia; 5 Department of Immunology and Transfusion Medicine, St. Olavs University Hospital, Trondheim, Norway

**Keywords:** Medical research, Rheumatology

## Abstract

Persons with rheumatoid arthritis (RA) or diabetes have increased risk of cardiovascular disease (CVD) and higher death rates compared to the general population. This study used data from the population-based Nord-Trøndelag Health Study (HUNT) and the Norwegian Cause of Death registry to compare all-cause mortality rates for RA or diabetes patients to the general population. We used Cox regression with age as time variable, adjusting for sex, smoking, body mass index, hypertension, total cholesterol, creatinine and previous CVD. To achieve proportional hazards, an interaction term with an age group variable (≤75 years or >75 years) was included for diabetes, smoking and previous CVD. Median follow-up was 18.1 years. Mortality occurred for 123 (32%) of the RA patients, 1,280 (44%) of the diabetes patients, 17 (52%) of the patients with both diseases and 11,641 (18%) of the controls. Both diseases were associated with statistically significantly increased mortality rates. The hazard ratio (HR) for RA was 1.24 (95% CI: 1.03-1.44). The HR of diabetes was 1.82 (1.60-2.04) for individuals ≤75 years old and 1.49 (1.39-1.59) for individuals >75 years. Diabetes had a significantly higher HR for death than RA for participants ≤75 years, but not significantly different for participants >75 years.

## Introduction

Rheumatoid arthritis (RA) is a systemic inflammatory disease causing inflammation in the synovia. It may lead to joint destruction and extra-articular manifestations such as pericarditis, vasculitis, osteoporosis, rheumatoid nodules and Sjögren’s syndrome^1^. RA patients have increased risk of cardiovascular events, as well as have higher cardiovascular, respiratory, and all-cause mortality rates compared to the general population^2–10^.

The risk of cardiovascular disease (CVD) associated with RA has been compared to that of diabetes, a well-known risk factor for CVD and premature death^11–13^. Recent evidence indicates that patients with either disease have increased risk of CVD compared to the general population, however, RA was associated with a lower increase in risk than diabetes^14^. This somewhat contradicts earlier studies indicating comparable risk in the two groups, and might be explained by changes in treatment of RA and differences in study populations^3,15,16^.

In the past decades, the general population in the developed world has become healthier and lives longer^17–19^. This trend also seems to apply to patients with diabetes^20^. However, for RA patients the results over time are more conflicting, from widening mortality gap to better survival than the general population^2,4,5,7,21–28^. These contradictory findings might be explained by differences in genetics, demographics and health as well as differences in inclusion of incident or prevalent cases, in RA definition, and in follow-up time. However, most evidence based on large numbers of patients and long observation periods points towards lower absolute mortality rates among RA patients in recent years, though still higher than in the general population^4,5,7,24–26^.

All-cause mortality among RA patients has been compared to that of diabetes patients in a perioperative setting^29^. However, to our knowledge it has not been compared in a longitudinal study in a general population, which would extend the implications of the findings. Awareness about the increased mortality risk associated with diabetes is high, whereas the increased risk associated with RA is less commonly considered by non-rheumatologists. Direct comparison would likely be of interest to patients and clinicians. Data from the longitudinal Nord-Trøndelag Health Study (HUNT) linked to the Norwegian Cause of Death Registry are ideal for such a comparison with detailed participant information and long follow-up time^30,31^.

The hypothesis was that RA is a risk factor for increased mortality rates not significantly different in magnitude from diabetes, and that the percentage of deaths caused by CVD were increased in both patient groups. The primary aim of the study was to compare all-cause mortality rates in patients with RA and patients with diabetes to that of the general population. The secondary aim was to evaluate whether the causes of death differed among the groups.

## Materials and Methods

All methods were carried out in accordance with relevant guidelines and regulations.

### Participants and variable definitions

**Figure 1:**
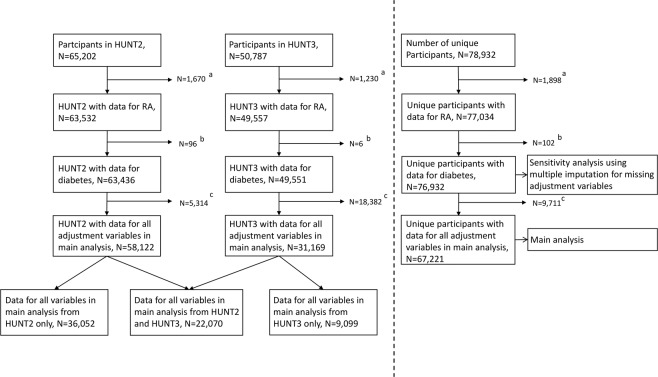
Participant inclusions and exclusions. Abbreviations: HUNT2 – second survey of Nord-Trøndelag Health Study, HUNT3 – third survey of Nord-Trøndelag Health Study, RA – rheumatoid arthritis. ^a^Exclusions due to uncertainty regarding RA status, or presence of psoriasis arthritis, juvenile inflammatory arthritis, ankylosing arthritis or other forms of inflammatory arthritis. ^b^Exclusions due to missing data to evaluate diabetes status. ^c^Exclusions due to missing data regarding smoking, hypertension, previous cardiovascular disease, creatinine concentration, total cholesterol concentration or body mass index.

This study utilized data from HUNT, a longitudinal population-based health study that invited all inhabitants of the Norwegian region Nord-Trøndelag aged ≥20 years to participate^30^. HUNT was designed to investigate the epidemiology of common diseases and quality of life in the general population with the first survey in 1984–1986. New surveys have been conducted with 11-year intervals, also including new individuals as they became adults. The present study included data from the second and third survey; HUNT2 and HUNT3. HUNT2 (1995–1997) had 65,202 participants (69.5% of those invited) and HUNT3 (2006–2008) had 50,787 participants (54.1% of those invited). Out of these, 37,056 individuals participated in both HUNT2 and HUNT3. Figure 1 shows inclusions and exclusions to the present study.

HUNT collected data from questionnaires, non-fasting blood sample and physical examination, as previously described^30^. Subjects were classified as having RA and/or having diabetes, or being controls. There are many false-positive RA diagnoses in self-reported surveys. Therefore, we applied validated RA diagnoses from a previous study^32^. In that study, hospital case notes of participants self-reporting to have RA in HUNT were reviewed and RA status, time of diagnosis, and status for immunoglobulin M rheumatoid factor and anti-citrullinated protein antibody (ACPA) were recorded. RA was defined according to American College of Rheumatology/European League Against Rheumatism (ACR/EULAR) 2010 criteria^33^, documented in hospital case notes before the person participated in HUNT. With insufficient information to evaluate ACR/EULAR 2010 criteria, a previous diagnosis based on the ACR 1987 revised criteria qualified^34^. Seropositivity was defined as a positive test for rheumatoid factor or ACPA. We excluded participants with missing data to evaluate RA status, uncertainty whether the diagnosis was given after participation in HUNT, and patients with juvenile inflammatory arthritis, psoriasis arthritis, ankylosing spondylitis, or other forms of inflammatory arthritis (Fig. 1).

Diabetes was defined as self-reported diabetes, use of anti-diabetic medication, or having a non-fasting blood glucose level >11.1 mmol/L. Validation in HUNT1 found self-reported diabetes to be highly reliable compared to the patients’ hospital case notes, with a positive predictive value of 96.1% and a negative predictive value of 99.7%^35^. In the present study, we further increased the sensitivity of the definition as participants with high non-fasting blood sugar level or reporting to use anti-diabetics also were classified as having diabetes. Those fulfilling these criteria in HUNT2, and not in HUNT3, were assumed to still have diabetes in HUNT3 (N = 6). We excluded participants who lacked information to evaluate diabetes status. The remaining participants, who did not fulfil the criteria for RA or diabetes, served as controls.

Hypertension was defined as systolic blood pressure ≥140 mmHg, diastolic blood pressure ≥90 mmHg or being on blood pressure-lowering medication. Smoking habits were classified as never, former or current smoker. Previous CVD was defined as self-reported angina, stroke, or myocardial infarction. Body mass index (BMI) was categorized into 5 groups: <18.5 kg/m², 18–24.9 kg/m², 25–29.9 kg/m², 30–34.9 kg/m² and ≥35 kg/m².

The baseline of each participant was their first participation in HUNT2 or HUNT3. For those who participated in both surveys, the first observation with complete data on the variables included in the main analysis was considered the baseline observation. Hence, for those participating in both HUNT2 and HUNT3, HUNT3 was defined as baseline if there were missing data in HUNT2 and not in HUNT3 (N = 1,371). If a participant did not have any observations in HUNT with complete data for the variables included in the main analysis their baseline was set to the first time they participated.

Most baseline adjustment variables had <4% missing data, with some exceptions. Data on smoking was missing for 3,176 (8%) and 1,970 (13%) with HUNT2 and HUNT3 as baseline, respectively. Blood pressure measurement data were missing for 1,900 (13%) with baseline in HUNT3. Data for creatinine were lacking for 3,776 (27%) with baseline in HUNT3. We excluded participants with incomplete information for at least one observation on all adjustment variables used in the final model (Fig. 1).

The data from HUNT were linked with the Norwegian Cause of Death Registry^31^. The registry includes information regarding all deaths in Norway and death of Norwegian citizens abroad. The International Classification of Diseases revision 10 (ICD-10 or converted from ICD-9) codes listed as the underlying cause of death were used to evaluate causes of deaths. Among the participants who died, 110 (0.9%) lacked an ICD code (diabetes patients: n = 9, controls: n = 101). Participants were followed up from their baseline observation in HUNT until death or end of observation on 31.12.2014, whichever came first.

### Statistical analysis

Data are given as medians with 25^th^ and 75^th^ percentiles, means with standard deviation, or numbers with percentage. Statistical analyses were performed using Stata (v. 15.1, StataCorp, College Station, TX, USA). P-values < 0.05 were considered statistically significant.

RA and diabetes were coded as yes/no variables; thus, those with both diseases were not coded as a separate group due to low number (N = 33). Kaplan-Meier survival curves were made to visualize survival in the groups. The few participants with both diseases were excluded from the plot.

Survival was evaluated using Cox proportional hazard regression modelling. Age was used as the time variable, with entry date at the baseline observation. This design ensured that participants were compared to other participants of the same age in all models. The alternative approach using time of diagnosis as the start of observation period was considered inappropriate. Firstly, there was no equivalent date for the controls. Secondly, age at time of diagnosis was self-reported for diabetes patients and collected from medical records in RA patients. This leads to a potential recall bias for the diabetes patients and a systematic error due to different data collection methods.

The proportional hazards (PH) assumption was assessed with Schoenfeld residuals, Cox-Snell residuals and log-minus-log plots. In order to meet the assumption, we included an interaction term between variables not initially fulfilling the assumptions and an age group variable; ≤75 years and >75 years. Participants turning 75 years in the observation period belonged to the first age group until they turned 75, and then changed to the second age group. The models were compared using log likelihood, Akaike information criteria and Bayesian information criteria. For subjects participating twice, data were updated at their second participation. The disease effects of RA and diabetes were compared by evaluating confidence intervals (CI) of the hazard ratios (HR).

Cox regression modelling was performed in three steps. Age was the time variable and thereby adjusted for in all models. On Step 1, the crude effects of RA and diabetes were investigated, with adjustment for sex. On Step 2, further adjustment for variables important for mortality was performed, i.e. hypertension, smoking, BMI, creatinine, total cholesterol. Cause-specific mortality rates attributed to CVD are elevated in RA and diabetes patients, compared to in the general population^2,6,7,12^. On Step 3, the analysis was therefore further adjusted for previous CVD. The main models included an interaction term between diabetes and the age group variable to meet the PH assumption, resulting in separate HR for diabetes patients ≤75 years and >75 years. Similarly, an interaction term with age group for smoking and previous CVD were also included. To avoid loss of statistical power to fewer deaths and because the PH assumption was not violated for RA, an interaction with age groups was not included for RA. In Sensitivity analysis 1, the analyses were repeated including the interaction term with age groups both for RA and diabetes.

In Sensitivity analysis 2, the Cox models were run following stratification by birth year into 10-year intervals instead of including the interaction term with age ≤75 years or >75 years. This approach provides the average HR over all age strata for RA and diabetes compared to controls, while permitting the baseline hazard to vary among strata. In Sensitivity analysis 3, the Step 1 models were run separately for diabetes and controls in each 10-year stratum. Similar analysis could not be run for RA due to fewer deaths.

Missing data may introduce bias in analyses only including complete cases. Sensitivity analysis 4 was performed to investigate the robustness of the result, using multiple imputation of missing data in adjustment variables. Multiple imputation (n = 50 datasets) was performed using chained equations assuming missing at random.

In the main analysis HR were calculated for each disease variable. Participants with both diseases contributed to the HR both for RA and diabetes, because the number with both diseases was considered too low to group them separately. To investigate whether this group influenced the result of the main models, Sensitivity analysis 5 was a Step 3 model excluding participants with both diagnoses. The importance of missing data for diabetes medication or report of no diabetes treatment was investigated in Sensitivity analysis 6, adding a categorical variable indicating treatment/no treatment/missing information on medication to a Step 1 model.

The causes of death were evaluated by comparing the CI of the percentage estimates, which were calculated based on the Poisson distribution.

### Ethics

Participants of HUNT gave written informed consent. Approval for the study was obtained from the Regional Committee on Medical Research Ethics, Central Norway (project 2009/661), the Norwegian Data Safety Authorities and the Norwegian Department of Health.

## Results

### Study population

In total 67,221 participants had complete data for at least one time point, i.e. either HUNT2 or HUNT3 (Fig. 1). There were 420 RA patients, 2,931 diabetes patients and 63,903 controls. Among the patients 33 individuals had both RA and diabetes. Baseline participant characteristics are given in Table 1 and disease-specific characteristics are given in Table 2.

**Table 1:** Baseline characteristics – cases with complete data^a,b,c^.

	RA N = 387	Diabetes N = 2,898	RA + Diabetes N = 33	Controls N = 63,903
Sex				
Female	261 (67)	1,369 (47)	20 (61)	33,781 (53)
Male	126 (33)	1,529 (53)	13 (39)	30,122 (47)
Age (years)	58 (49, 68)	63 (51, 73)	66 (59, 73)	46 (34, 60)
Smoking status				
Never smoker	132 (34)	1,263 (44)	17 (52)	29,426 (46)
Former smoker	129 (33)	682 (23)	4 (12)	19,010 (30)
Current smoker	126 (33)	953 (33)	12 (36)	15,467 (24)
Previous CVD^d^	41 (11)	594 (20)	7 (21)	4,094 (6)
Body mass index (kg/m²)	26.0 (23.7, 29.1)	28.7 (25.9, 32.0)	27.8 (24.4, 30.9)	25.7 (23.4, 28.4)
Hypertension^e^	199 (51)	2,098 (72)	26 (79)	25,333 (40)
Waist/hip ratio	0.84 (0.79, 0.90)	0.90 (0.84, 0.95)	0.88 (0.83, 0.93)	0.85 (0.79, 0.90)
Non-fasting glucose (mmol/L)	5.2 (4.8, 5.9)	7.5 (5.8, 11.2)	6.7 (5.6, 8.1)	5.1 (4.7, 5.6)
Creatinine (µmol/L)	84 (77, 92)	89 (80, 99)	89 (80, 98)	85 (77, 94)
Triglycerides (mmol/L)	1.4 (1.0, 2.0)	2.1 (1.4, 3.0)	1.5 (1.2, 2.2)	1.4 (1.0, 2.1)
Total cholesterol (mmol/L)	6.0 (5.3, 6.8)	6.0 (5.1, 6.9)	5.8 (5.0, 6.8)	5.7 (4.9, 6.5)
HDL cholesterol (mmol/L)	1.4 (1.1, 1.7)	1.2 (1.0,1.4)	1.4 (1.1, 1.7)	1.3 (1.1, 1.6)
Total cholesterol/HDL cholesterol ratio	4.3 (3.5, 5.5)	5.1 (4.0, 6.5)	4.3 (3.6, 5.4)	4.2 (3.4, 5.3)
Observation time (years)	18.1 (12.6, 18.8)	17.1 (7.6, 18.6)	16.8 (8.9, 18.2)	18.1 (12.7, 18.8)

^a^Number (%) or median (25^th^ and 75^th^ percentile) unless specified otherwise.

^b^Abbreviations: RA - rheumatoid arthritis, CVD - cardiovascular disease, HDL - high-density lipoprotein.

^c^Missing data: <4% missing for all variables.

^d^Previous cardiovascular disease: self-reported angina, stroke, or myocardial infarction.

^e^Hypertension: Systolic blood pressure ≥140 mmHg, diastolic blood pressure ≥ 90 mmHg, or on blood pressure-lowering medication.

**Table 2:** Disease-specific baseline variables^a,b,c^.

	RA N = 387	Diabetes N = 2,898	RA + Diabetes N = 33
**RA-specific baseline variables**			
Seropositive^d^	277 (72)		24 (73)
Age when diagnosed (years)	55 (45, 64)		60 (53, 70)
RA duration before first HUNT participation with diagnosis (years)	6 (3, 9)		7 (5, 10)
**Diabetes-specific baseline variables**			
Peroral diabetes medication		1,091 (38)	11 (33)
Insulin		597 (21)	10 (30)
Age when diagnosed (years)		59 (48, 67)	64 (49, 69)
Diabetes duration before first HUNT participation with diagnosis (years)		5(2, 11)	6 (4, 16)

^a^Number (%) or median (25th and 75th percentile) unless specified otherwise.

^b^Abbreviations: RA - rheumatoid arthritis, HUNT – Nord-Trøndelag Health Study.

^c^Missing data: <4% missing for all variables except the diabetes-specific variables: peroral diabetes medication use missing for 755 (26%), insulin use missing for 761 (26%), duration of diabetes and age of diabetes onset missing for 192 (7%).

^d^Seropositive: positive for rheumatoid factor and/or anti-citrullinated protein antibody.

Participants with diabetes were slightly older than those with RA, and as a group controls were younger than diabetes and RA patients. There were more females in the RA group, and more RA patients were ever smokers. Diabetes patients more often had hypertension, had higher median BMI, waist/hip ratio, total cholesterol/HDL-cholesterol ratio, concentration of creatinine, glucose and triglycerides, and lower concentrations of HDL cholesterol. RA and diabetes patients had higher prevalences of previous CVD compared to controls, highest among diabetics. Baseline and disease-specific characteristics of participants in the first sensitivity analysis are presented in Supplementary Table S1.

### Mortality

Throughout the follow-up period, 123 participants (32%) with RA, 1,280 participants (44%) with diabetes, 17 participants (52%) with RA and diabetes and 11,641 controls (18%) died. Total observation time was 1,034,596 person years; 5,999 person years in the RA group, 38,760 person years in the diabetes group, 445 person years in the RA and diabetes group, and 989,392 person years in the control group.

**Figure 2:**
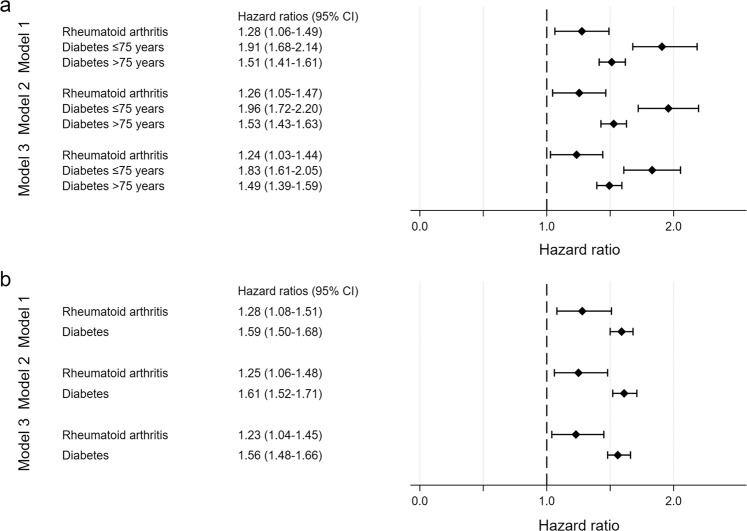
Cox regression models for mortality in rheumatoid arthritis and diabetes. Panel a: Main models. Panel b: Sensitivity analysis 2 – stratified by birth year intervals. Age was used as time axis in all models and is thereby adjusted for in all models. Model 1: Adjusted for sex. Model 2: Adjusted for sex, hypertension^1^, smoking^2^, BMI^3^, creatinine, total cholesterol. Model 3: Adjusted for sex, hypertension^1^, smoking^2^, BMI^3^, creatinine, total cholesterol, previous cardiovascular disease^4^. Abbreviations: 95% CI – 95% confidence interval, BMI – body mass index. ^1^Hypertension: systolic blood pressure ≥ 140, diastolic blood pressure ≥ 90, or on blood pressure-lowering medication. ^2^Smoking: never smoker, former smoker or current smoker. ^3^BMI: categorised as <18.5 kg/m², 18–24.9 kg/m², 25–29.9 kg/m², 30–34.9 kg/m² and ≥35 kg/m². ^4^Previous cardiovascular disease: self-reported angina, stroke or myocardial infarction.

RA and diabetes were significantly associated with increased mortality compared to controls, and the association remained significant after adjustment for other variables. Figure 2a gives the results from the main Cox regression analysis calculating separate HR for the diabetes patients ≤75 and >75 years of age. The HR for death in patients with diabetes was higher for participants ≤75 years of age than for those >75 years of age. The HR estimates for diabetes patients in both these age groups were higher than for RA patients. However, the difference was only significantly different for the diabetes patients >75 years because the CI for the diabetes patients <75 years were overlapping with those of the RA patients.

In Sensitivity analysis 1, the same age grouping was used for RA. The HR for RA compared to controls for patients ≤75 years and patients >75 were very similar to the HR without age grouping from to the main models, but the CI were wider due to fewer events in each group, especially in the lower age group. (Supplementary Table S2).

**Figure 3:**
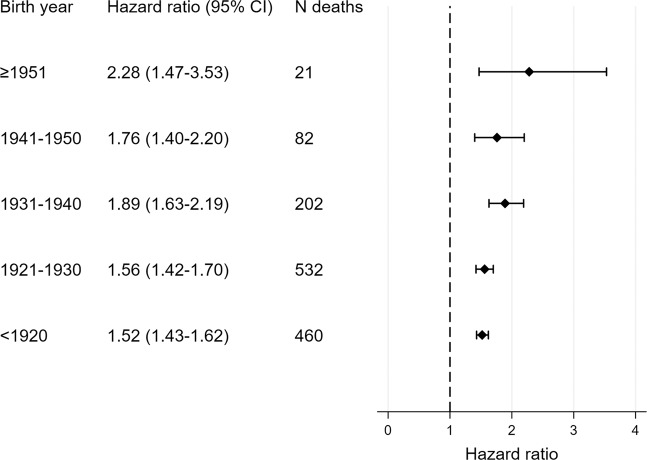
Cox regression models for mortality in diabetes according to birth year stratum. Age was used as time axis in all models and is thereby adjusted for in all models. Models were also adjusted for sex. Abbreviations: 95% CI – 95% confidence interval. Participants born in 1951 or later were pooled due to few deaths in the younger strata.

In Sensitivity analysis 2 stratified by birth year in 10-year intervals, the results for RA were very close to those from the main analysis at all three steps, as expected due to the PH (Fig. 2b). The HR for diabetes were between the estimates for the two age groups in the main model. Diabetes had significantly higher HR for mortality than RA at all steps. In Sensitivity analysis 3 a separate Step 1 model was run for diabetes and controls for each age stratum. The results confirmed that the HR for mortality tended to be lower in the older age strata, but the CI were much wider in the younger age strata due to fewer deaths (Fig. 3).

**Figure 4:**
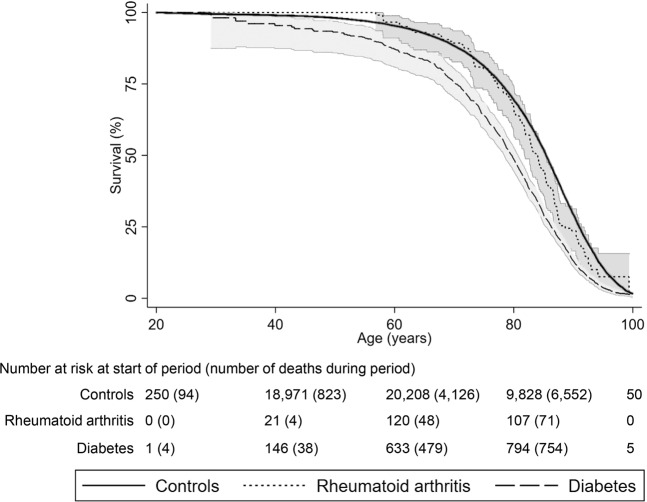
Kaplan-Meier survival curves. Kaplan-Meier survival estimates with 95% confidence intervals for rheumatoid arthritis patients, diabetes patients and controls. Participants with both diseases not presented.

Figure 4 shows Kaplan-Meier survival estimates.

Sensitivity analysis 4 using multiple imputation of missing adjustment variables gave similar results as the main analysis, with essentially unchanged HR (Supplementary Table S2). Sensitivity analysis 5, in which the participants with both diseases were excluded in the Step 3 model, also resulted in essentially unchanged findings (Supplementary Table S2). Use of diabetes medication or not, or missing treatment information for diabetes patients (Sensitivity analysis 6), was not significant in the Step 1 model (p = 0.66).

**Figure 5:**
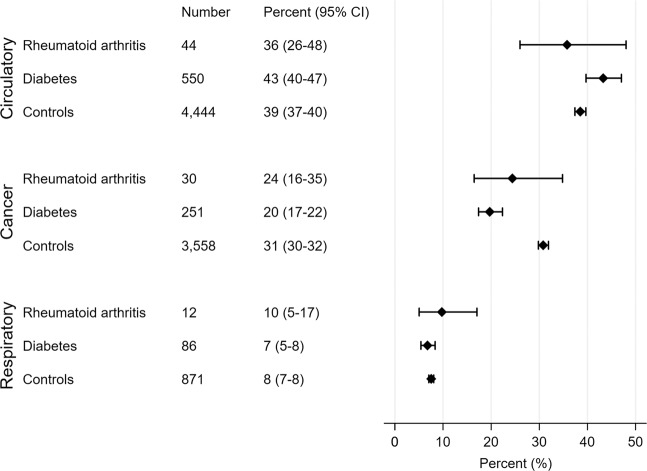
Distribution of causes of death. The number and percentages that died from each of the three main causes of deaths, for rheumatoid arthritis patients, diabetes patients and controls. Patients with both diseases not presented. Abbreviations: 95% CI – 95% confidence interval.

The three main causes of death were diseases of the circulatory system (ICD10 I00-I99), neoplasms (ICD10 C00-D49) and diseases of the respiratory system (ICD10 J00-J99). Figure 5 shows the distribution of deaths from three main causes among patients with either RA or diabetes and controls. The third major cause of death in the diabetes group was diabetes-related (ICD10 E10-E14), accounting for 12% of the deaths in this group. Among the patients with diabetes, a significantly higher percentage died from circulatory disease and a significantly lower percentage died from cancer compared to controls.

## Discussion

The main finding of this study was that both diabetes and RA were associated with increased mortality rates. For diabetes, the HR tended to be higher in the younger participants as defined by 10-year strata by birth year. The average HR for death was significantly higher for diabetes than for RA, and significantly higher for participants ≤75 years when diabetes patients were grouped with a cut-off at 75 years. The increased death rates could not be explained by the lifestyle factors and other health parameters adjusted for in the analysis.

As expected, the main of causes of death in were circulatory disease, neoplasms, and respiratory disease in that order. For the patients with diabetes, diabetes-related deaths was the third main cause of death and respiratory diseases the fourth. Overall death rates were higher for patients with diabetes and/or RA, which explains how one may find higher death rates from a specific cause, but lower percentage of deaths due to that cause. The percentages of different death causes agree well with previous reports^6,7,9–12,22^. The controls were younger, so the interpretation of the comparison of death causes between the groups need some caution.

The main analysis and Sensitivity analysis 3 confirmed that the mortality risk among diabetes patients compared to controls was highest in the younger age group. This is consistent with a nationwide Swedish study, reporting that the HR for death among diabetics compared to healthy matched controls attenuated with age^12^. They reported an adjusted HR during or after 2005 of 2.59 (95% CI: 2.27–2.29) for those <55 years and of 1.03 (95% CI: 1.01–1.03) for those ≥75 years, hence a lower HR estimate than the present study for patients ≥75. The higher HRs in the younger diabetes patients might be due lower expected death rates in the general population, or a survival bias; those with most severe diabetes die earlier. Furthermore, disease with onset earlier in life might be more severe and cause more comorbidities over time. A similar increase in HR in the younger age group was not found for RA. This could be due to differences between RA and diabetes, such as the distribution of risk factors and comorbidities, or differences related to delay from disease onset to diagnosis. However, because a previous study reported higher relative risk in younger compared to older RA patients, the present study might lack the power to detect such a difference^7^.

Previous studies have shown that all-cause death rates and cardiovascular-specific death rates are higher among patients with RA or diabetes than the general population^2,4,11,12^. There has been a focus on their risk of CVD, and on how to manage these patients^3,15,36,37^. A nationwide Danish study reported that the risk of myocardial infarction was comparable for diabetes and RA patients, with adjusted incidence rate ratios of 1.7 for both groups^3^. A large study from the US found that the risks of CVD were increased in both groups, although diabetes patients had a significantly greater risk than RA patients^14^. However, the magnitude, nature and consequence of a myocardial infarction may differ between the groups. In one study, RA patients had increased risk of silent ischemic heart disease, sudden death, and more often died shortly after developing heart failure compared to controls^38^. Another study from Taiwan found that RA patients more often than controls experienced adverse outcomes, including ischemic events and in-hospital mortality, following an acute cardiovascular event^39^.

There have been contradictory results regarding mortality trends among RA patients in the new millennium; from reports of increasing mortality gap, to reports of lower rates than the general population, to the more common reports of a reduction in absolute mortality rates though still higher rates than in the general population^4,7,21–24,27,28^. There has been a shift in treatment of RA patients with more emphasis on early intensive treatment, which has resulted in improved disease control^40–43^. Some of the RA specific-medications reduce CVD risk, and might contribute to reducing absolute mortality rates in RA patients^44–46^.

Most large previous studies were registry-based and did not have information for smoking, BMI etc., thus, other factors may have acted as confounders. Smoking is an acknowledged risk factor for RA as well as for CVD and early death. Nevertheless, adjusting for these variables in the present study only changed the estimates slightly.

The strength of our study is the population-based design, the large registers, and a reasonably high participation approximately 70% in HUNT2 and 50% in HUNT3^30^. The HUNT study is considered as fairly representative for the Norwegian population^30^, and there was a large number of relevant controls from the same population as the patients. We also had data not available from registries, like smoking habits and BMI. Another advantage is the long follow-up time, and that there were two surveys, making it possible to record changes over time.

Further, the RA diagnoses were validated from medical records, and can be considered accurate^32^. Data for vital status at the end of the observation period and date of death do also have very high accuracy. In contrast to previous studies where RA and diabetes have been compared with regards to perioperative mortality mortality^29^, this longitudinal study format gives more information from a long-term perspective. Direct comparison of RA and diabetes, which both are associated with increased mortality rates, is useful when evaluating the importance of risk factors influencing overall health and longevity and planning appropriate interventions.

Our study does have some limitations. It may be argued that introducing the age group variable for diabetes and not for RA in the main analysis might bias the results. The sensitivity analyses demonstrated that the main models were acceptable and that they captured an important difference in diabetes mortality depending on age, even if the exact cut-off age should be interpreted cautiously. A similar age difference was not present for RA.

We were not able to distinguish between type I and type II diabetes. A Public Health Report estimated that there were 28,000 type I diabetes patients and 216,000 type II diabetes patients in Norway in 2014^47^. Thus, the majority in HUNT would have type II. Further, some participants may have developed RA or diabetes following their participation in HUNT. They would be controls in the present study, which could give an underestimation of the effect of RA or diabetes on mortality. The large number of controls makes that less likely.

Use of diabetes medication or not could potentially influence mortality rates in diabetes patients, but many participants had not answered the relevant questions. The sensitivity analysis indicated that treatment/no treatment/missing information was not associated with mortality in this patient group.

Further, there might have been a participation bias in HUNT. The proportion of participants among those invited was approximately 70% in HUNT2 and 50% in HUNT3^30^. A study of non-participants showed that they had reduced survival, lower socioeconomic status, higher prevalence of chronic diseases and higher proportion with diabetes than participants, though musculoskeletal pain was less common among non-participants^48^. If the sickest RA and diabetes patients did not participate, HR would be underestimated. Nevertheless, it is likely that such a bias would extend to the controls, hence not be excessive.

The adjustment variables in the models were registered at participation in HUNT. Ideally, they should have been registered before onset of disease, which was not possible due to HUNT’s design. Some of the adjustment variables may act as mediators of mortality. To avoid drawing wrong conclusions due to erroneous adjustments the analyses were performed in a stepwise fashion with adjustment only for age and sex on Step 1. The study was not designed to identify mediators of the increased mortality in RA and diabetes, but the changes in HR from Step 1 to Step 3 in the main models were very small. We cannot exclude that other schemes for registration of adjustment variables, e.g. before diagnosis for all participants or with more frequent updates, could alter the results.

The self-reported data from HUNT have some uncertainty, particularly for smoking status and previous CVD. The underlying causes of death are in nature a bit uncertain and the low number of RA patients increased the uncertainty for their distribution of death causes.

In conclusion, the risk of death was higher for patients with either RA or diabetes compared to the general population. Diabetes patients had a significantly higher HR for death than RA patients for individuals 75 years of age or younger, but not for patients older than 75 years.

## Supplementary information

Supplemental information.

## Data Availability

Data from HUNT are available upon reasonable request from the HUNT Research Centre (https://www.ntnu.edu/hunt/data), following approval from the Regional Research Ethics Committee. However, restrictions apply to the availability of the data for the present paper, which were used under license for the current study as detailed above (Ethics section) and are not publicly available in accordance with Norwegian law.
