# Supplementary material for: Mortality is increased in patients with rheumatoid arthritis or diabetes compared to the general population – the Nord-Trøndelag Health Study
Source: Sci Rep. 2020 Feb 27;10:3593. doi: 10.1038/s41598-020-60621-2 (PMC7046618; doi:10.1038/s41598-020-60621-2)
Supplement: Supplementary file 1 — Supplemental information. [file 41598_2020_60621_MOESM1_ESM.pdf]

SUPPLEMENTARY FILE

Mortality is increased in patients with rheumatoid arthritis or diabetes compared to the general population – the Nord-Trøndelag Health Study

Ingrid Sæther Houge [1], Mari Hoff [2,3], Ranjeny Thomas [4], Vibeke Videm* [1,5]

[1]Department of Clinical and Molecular Medicine, NTNU - Norwegian University of Science and Technology, Trondheim, Norway

[2]Department of Public Health and Nursing, NTNU – Norwegian University of Science and Technology, Trondheim, Norway

[3]Department of Rheumatology, St. Olavs University Hospital, Trondheim, Norway

[4]Diamantina Institute, University of Queensland, Brisbane, Australia

[5]Department of Immunology and Transfusion Medicine, St. Olavs University Hospital, Trondheim, Norway

*Vibeke Videm, MD, PhD

Department of Clinical and Molecular Medicine, Lab Center 3 East

St. Olavs University Hospital, NO-7006 Trondheim, Norway

Tel: +47 72 57 33 21, Fax: +47 72 27 64 26, e-mail: vibeke.videm@ntnu.no

Supplementary Table S1: Baseline and disease-specific characteristics including participants with missing covariates [1,2,3]

	RA N=475	Diabetes N=3,820	RA + Diabetes N=41	Controls N=72,582
**Baseline characteristics**				
Sex				
Female	326 (69)	1,852 (48)	25 (61)	38,571 (53)
Male	149 (31)	1,969 (52)	16 (39)	34,024 (47)
Age (years)	58 (49, 68)	62 (51, 73)	66 (59, 73)	46 (34, 60)
Smoking status				
Never smoker	152 (32)	1,527 (40)	18 (44)	31,569 (43)
Former smoker	160 (34)	846 (22)	9 (22)	19,788 (27)
Current smoker	140 (29)	1,156 (30)	14 (34)	16,407 (23)
Previous CVD [4]	51 (11)	754 (20)	10 (24)	4,685 (6)
BMI (kg/m²)	26.1 (23.8, 29.0)	28.9 (26.1, 32.2)	27.8 (24.4, 30.9)	25.7 (23.4, 28.4)
Hypertension [5]	233 (49)	2,711 (71)	29 (71)	27,609 (38)
Waist/hip ratio	0.84 (0.79, 0.90)	0.90 (0.84, 0.96)	0.88 (0.84, 0.93)	0.85 (0.79, 0.90)
Non- fasting glucose	5.2 (4.7, 5.9)	7.3 (5.7, 10.7)	6.7 (5.5, 9.6)	5.1 (4.7, 5.6)

(mmol/L)				
Creatinine (µmol/L)	83 (76, 92)	89 (80, 99)	85 (79, 96)	85 (77, 94)
Triglycerides (mmol/L)	1.4 (1.0, 2.0)	2.1 (1.4, 3.1)	1.6 (1.3, 2.4)	1.4 (1.0, 2.1)
Total cholesterol (mmol/L)	5.9 (5.2, 6.8)	6.0 (5.2, 6.9)	6.0 (5.2, 6.8)	5.6 (4.8, 6.5)
HDL cholesterol (mmol/L)	1.4 (1.1, 1.7)	1.2 (0.9, 1.4)	1.3 (1.1, 1.6)	1.3 (1.1, 1.6)
Total cholesterol/HDL cholesterol ratio	4.2 (3.4, 5.4)	5.1 (4.1, 6.5)	4.5 (3.6, 5.5)	4.2 (3.3, 5.3)
Observation time (years)	18.0 (12.1, 18.8)	17.4 (7.5, 18.4)	14.6 (7.2, 18.2)	17.9 (8.0, 18.7)
**RA-specific variables**				
Seropositive [6]	348 (73)		31 (76)	
Age when diagnosed (years)	55 (44, 65)		61 (53, 70)	
RA duration before first HUNT participation with diagnosis (years)	6 (3, 9)		7 (4. 9)	
**Diabetes-specific variables**				
Peroral diabetes medication		1,469 (38)	15 (37)	
Insulin		722 (19)	10 (24)	
Age when diagnosed (years)		59 (49, 68)	61 (50, 69)	

Diabetes duration before first HUNT participation with diagnosis (years)	5 (2, 11)	6 (3, 12)

[1]Number (%) or median (25th and 75th percentile)

[2]RA: rheumatoid arthritis, CVD: cardiovascular disease, BMI: body mass index, HDL: high-density lipoprotein

[3]Missing data – <4% missing data except for smoking, blood pressure, creatinine, triglycerides and diabetes-specific variables. Smoking missing for 3,176 (5 %) and 1,970 (13%) with baseline in HUNT2 and HUNT3, respectively. Blood pressure missing for 1,900 (13%) with baseline in HUNT3. Creatinine and triglycerides missing for 3,777 (25%) with baseline in HUNT3. Diabetes-specific variables: peroral diabetes medication use missing for 1,058 (27%), insulin use missing for 1,072 (28 %), duration of diabetes and age of diabetes onset missing for 299 (8%).

[4]Previous cardiovascular disease: self-reported angina, stroke, or myocardial infarction

[5]Hypertension: Systolic blood pressure ≥ 140, diastolic blood pressure ≥ 90, or on blood pressure-lowering medication

[6]Seropositive – positive for either rheumatoid factor and/or anti-citrullinated protein antibodies

Supplementary Table S2: Sensitivity analyses 1, 4, and 5

	Hazard ratio	95 % Confidence interval	P-value
**Sensitivity analysis 1: Cox regression for mortality by age groups**			
Rheumatoid arthritis ≤75 years	1.21	0.79-1.62	0.25
Rheumatoid arthritis >75 years	1.24	1.01-1.48	0.035
Diabetes age ≤75 years	1.83	1.61-2.05	<0.001
Diabetes age >75 years	1.49	1.39-1.59	<0.001
**Sensitivity analysis 4: Cox regression for mortality after multiple imputation of missing adjustment variables**			
Rheumatoid arthritis	1.20	1.03-1.40	0.021
Diabetes age ≤75 years	1.90	1.70-2.12	<0.001
Diabetes age >75 years	1.43	1.27-1.62	<0.001
**Sensitivity analysis 5: Cox regression for mortality excluding patients with both diseases**			
Rheumatoid arthritis	1.24	1.02-1.46	0.017
Diabetes age ≤75 years	1.83	1.61-2.06	<0.001
Diabetes age >75 years	1.52	1.39-1.59	<0.001

All models are from Step 3, i.e. adjusted for sex, hypertension [1], smoking [2], BMI [3], creatinine, total cholesterol, and previous cardiovascular disease [4]

Age was used as the time variable and was thereby adjusted for in all models

Abbreviations: BMI – body mass index

[1]Hypertension: Systolic blood pressure ≥ 140, diastolic blood pressure ≥ 90, or on blood pressure-lowering medication

[2]Smoking: never smoker, current smoker or former smoker

[3]BMI: categorised as <18.5 kg/m², 18.5-24.9 kg/m², 25-29.9 kg/m², 30-34.9 kg/m² and ≥35 kg/m²

[4]Previous cardiovascular disease: self-reported angina, stroke, or myocardial infarction
